# Prediction of pathological activity in Crohn’s disease based on dual-energy CT enterography

**DOI:** 10.1007/s00261-024-04276-x

**Published:** 2024-04-10

**Authors:** Hai-fei Zhou, Wei Chen, Jing-qi Li, Gen-ji Bai, Li-li Guo

**Affiliations:** 1 Department of Radiology, The Affiliated Huai’an NO.1 People’s Hospital of Nanjing Medical University, 1# Huanghe West Road, Huaiyin District, Huai’an, 223300 Jiangsu Province China; 2 Department of Pathology, The Affiliated Huai’an NO.1 People’s Hospital of Nanjing Medical University, 1# Huanghe West Road, Huaiyin District, Huai’an, 223300 Jiangsu Province China

**Keywords:** Crohn’s disease, Dual-energy, CT enterography, Pathological activity

## Abstract

**Purpose:**

To explore the feasibility of predicting the pathological activity of Crohn’s disease (CD) based on dual-energy CT enterography (DECTE).

**Methods:**

The clinical, endoscopic, imaging and pathological data of 55 patients with CD scanned by DECTE were retrospectively analyzed; the pathological results were used as a reference standard to classify the diseased bowel segments into active and inactive phases. The normalized iodine concentration (NIC), energy-spectrum curve slope K, dual energy index (DEI), fat fraction (FF) of the arterial phases and venous phases were compared. To assess the parameters’ predictive ability, receiver-operating characteristic curves were used. The Delong test was used to compare the differences between the diagnostic efficiency of each parameter.

**Results:**

A total of 84 intestinal segments were included in the study, including 54 active intestinal segments and 30 inactive intestinal segments. The NIC, energy-spectrum curve slope K and DEI were significantly different between active and inactive bowel segments in the arterial and venous phases (*P* < 0.05), while FF were not significantly different (*P* > 0.05). The largest area under the curve (AUC) of NIC, energy-spectrum curve slope K and DEI were higher in arterial phase than in venous phase. For identifying the intestinal activity of CD, the maximum AUC of NIC in arterial phase was 0.908, with a sensitivity of 0.833 and a specificity of 0.800, and the DEI in arterial phase had the highest sensitivity (0.944).

**Conclusion:**

The NIC, energy-spectrum curve slope K and DEI can effectively distinguish the active and inactive phases of the intestinal segments of CD patients and provide good assistance for determining further treatment.

**Graphical abstract:**

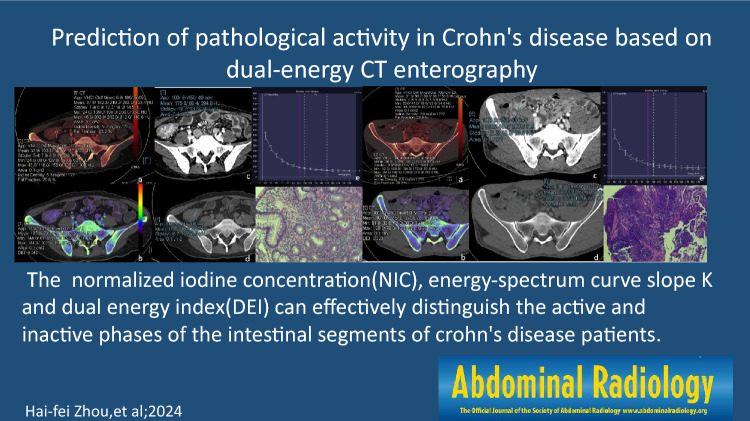

## Introduction

Crohn’s disease (CD) is a recurrent chronic nonspecific inflammatory disease that affects all parts of the gastrointestinal tract, with the terminal ileum, ileocecal region and adjacent colon being the most commonly involved, and associated lesions are often multinodular and jumpy. Its etiology and pathogenesis are still unclear and may be related to autoimmune and genetic factors. CD is characterized by a young age of onset, long duration of disease, and alternating periods of remission and activity [1]. The clinical manifestations lack specificity and mostly consist of abdominal pain, diarrhea, anal fistulas and abdominal masses. In recent years, the incidence of CD has been increasing yearly [2, 3]. Timely and accurate determination of the activity of CD is of great value for clinical diagnosis and prognosis. At present, the main methods for judging CD activity are the Crohn’s disease activity index (CDAI), endoscopic index, CT enterography (CTE), magnetic resonance index of activity (MaRIA) and biochemical markers (such as fecal calprotectin (FC) and C-reactive protein (CRP)). CDAI is one of the most commonly used clinical indicators, but it is highly subjective and not always correlated with endoscopic or radiological findings [4]. Endoscopy is the "gold standard" for assessing mucosal inflammation in CD [5], providing measures such as the simplified endoscopic score (SES-CD) and the CD endoscopic severity index (CDEIS), but it is invasive, has poor patient compliance, is not conducive to disease monitoring, and does not reveal submucosal muscle layers or extraintestinal lesions. CTE can only assess the morphology to determine disease activity and lacks specificity, and there is often a crossover between the morphological manifestations of active and inactive CD [6]. MaRIA has disadvantages such as long calculation times, high complexity and large errors in selecting the ROI. FC is a systemic indicator correlated with CD activity, and the correlation between FC and endoscopic and histological activity was better in the presence of colon involvement than in the presence of ileum involvement [7]. In recent years, dual-energy CTE (DECTE) has become an excellent way to accurately evaluate CD patients. However, most previous studies have used endoscopy, biochemical markers and clinical features as reference standards, while pathological results are rarely used. The aim of this study was to quantitatively assess the activity of patients with CD using DECTE parameters with pathological findings as a reference standard.

## Materials and methods

### Patients

This study was approved by the hospital ethical review committee; because of the retrospective nature of the study, informed consent was not needed. We collected 123 patients with clinical, endoscopic, imaging and pathologically confirmed diagnoses of CD in our hospital from May 2020 to 2023. Inclusion criteria: (1) the diagnostic criteria for Crohn’s disease recommended by the American Gastrointestinal Association Clinical Guidelines (2018) [8] and age older than 18 years; (2) complete clinical and DECTE data; (3) examination by colonoscopy or enteroscopy; and (4) interval between enterography and endoscopy less than 1 week. Exclusion criteria: (1) iodine allergy; (2) pregnancy; (3) severe liver and kidney insufficiency; and (4) lack of pathological specimens. It is important to note that we studied the difference between active and inactive segments of the diseased bowel.

### Pathological reference criteria

A pathologist with 10 years of experience who was blinded to clinical, radiologic and endoscopic data classified the disease enteritis into active chronic enteritis and inactive enteritis according to the Expert Guidelines for the Pathological Diagnosis of Inflammatory Bowel Disease in China [9]. Chronic enteritis refers to the infiltration of a large number of lymphocytes and plasma cells in the lamina propria of the mucosa, and mucosal structural abnormalities, including crypt structural changes, metaplastic changes, basal plasma cell increase, and inflammatory polyps. Active chronic enteritis/colitis refers to chronic enteritis with active changes, including cryptitis, crypt abscesses, erosions, ulcers, etc., and cryptitis refers to the infiltration of neutrophils into the crypt epithelium, while crypt abscess refers to the accumulation of neutrophils in the crypt cavity, often accompanied by the destruction of the crypt epithelium. Erosion is a tissue defect superficial to the mucosa, and deeper tissue defects are called ulcers. Inactive enteritis/colitis refers to chronic enteritis without active manifestations, without cryptitis, crypt abscesses, erosions, ulcers, etc.

### DECTE technique and parameter measurement

Preparation: Patients were asked to fast for at least 8 h prior to the examination, took approximately 2000 ml of 2.5% isotonic mannitol solution orally in divided doses 1.5 h before scanning, and were injected with scopolamine 10 mg intravenously over 10 min to inhibit bowel movement.

CT scan: Using a Siemens Definition Flash dual-energy CT with the patient in the supine position, plain scans were taken with the following scan parameters: tube voltage 120 kV, reference tube current 210 mAs, automatic mAs technology (Care Dose 4D), pitch 1.2, spherical tube rotation time 0.5 s/r, collimation width 128*0.6 mm, recombination layer thickness 0.75 mm, recombination interval 0.5 mm. Dual-energy enhancement scans were taken with the following parameters: tube A volage 150 kV, tube B voltage 100 kV, automatic mAs technology (Care Dose 4D), pitch 0.6, spherical tube rotation time 0.5 s/r,collimation width 128*0.6 mm, recombination layer thickness 0.75 mm, recombination interval 0.5 mm, high pressure injector, contrast tracer method, select the region of interest (ROI) at the level of the descending aorta above the septum, when the CT value reaches 100 HU, 5S automatically promote the arterial phase. The venous phase scan was performed 25 s after the end of the arterial phase scan. The contrast medium was iophorol (320 mgI/ml), injected at 1.21 ml/kg body weight at a flow rate of 3 ml/s, followed by 30–40 ml of saline at the same flow rate. The scan was performed from the top of the diaphragm to the inferior border of the pubic symphysis.

Image postprocessing: The 150 kVp and 100 kV sequence images of the scanned arterial phases and venous phases were transferred to the Syngo. via workstation. The Dual Energy program was selected, and an iodine map was generated with the "Liver VNC" module. The "single energy" module was then selected for energy spectrum analysis. The system automatically generates the energy spectrum curve, adjusts the KeV value, obtains the 40 and 100 keV CT values, and selects the Zeff/Rho module to obtain the DEI.

Parameter measurement: According to CD Montreal staging [10], colonoscopy is combined with imaging morphological changes to locate the specific site of the lesion, both of which correspond to each other. Two radiologists with 10 years of experience and blinded to the pathological results, colonoscopy and clinical information of the patient measured iodine values, FF, the iodine values at the corresponding level in the aorta, 40 and 100 keV CT values and DEI in the same ROI region. Each parameter was measured three times per person and averaged over an ROI area of approximately 0.1 cm^2^ while avoiding areas of air and blood vessels. The NIC was calculated as follows: NIC = iodine concentration_Lesional bowel disease_/Iodine concentration_aorta at same level_; slope of the energy-spectrum curve slope K = CT_40KeV_-CT_100KeV_/60 [11].

### Statistical analysis

SPSS 25.0 software was applied for data analysis. The Kolmogorov–Smirnov (K–S) test was used to test the normality of the measurement data. Normally distributed data are expressed as the mean ± standard deviation (x ± s), and nonnormally distributed data are expressed as the median and interquartile range (M (Q, R)). The intraclass correlation coefficient (ICC) was used to evaluate the agreement between the 2 physicians. The independent samples *t* test or Mann‒Whitney *U* test was used to compare the NIC, energy-spectrum curve slope K, FF and DEI of the active and inactive groups for statistical significance. Receiver operating characteristic (ROC) curves were plotted, and the areas under the curve (AUCs) were calculated. Delong test was used to compare the diagnostic efficiency of each parameter. Differences were considered statistically significant at *P* < 0.05.

## Results

A total of 55 patients were enrolled in the study, The patient characteristics are shown in Table 1. The 68 patients were excluded because of severe liver and kidney function (5 patients), iodine allergy (1 patient), and lack of pathological results (62 patients).

**Table 1:** Patient characteristics

Parameters	Contents	
Sex (male:female)	34:21	
Mean age	28 years (18–62 years)	
Diseased intestinal segment (84)	Colon	19 (22.6%)
	Rectoanal	7 (8.3%)
	Ileocecal	42 (50%)
	Small bowel	16 (19.1%)
Active bowel segments	54	
Inactive bowel segments	30	

**Fig. 1:**
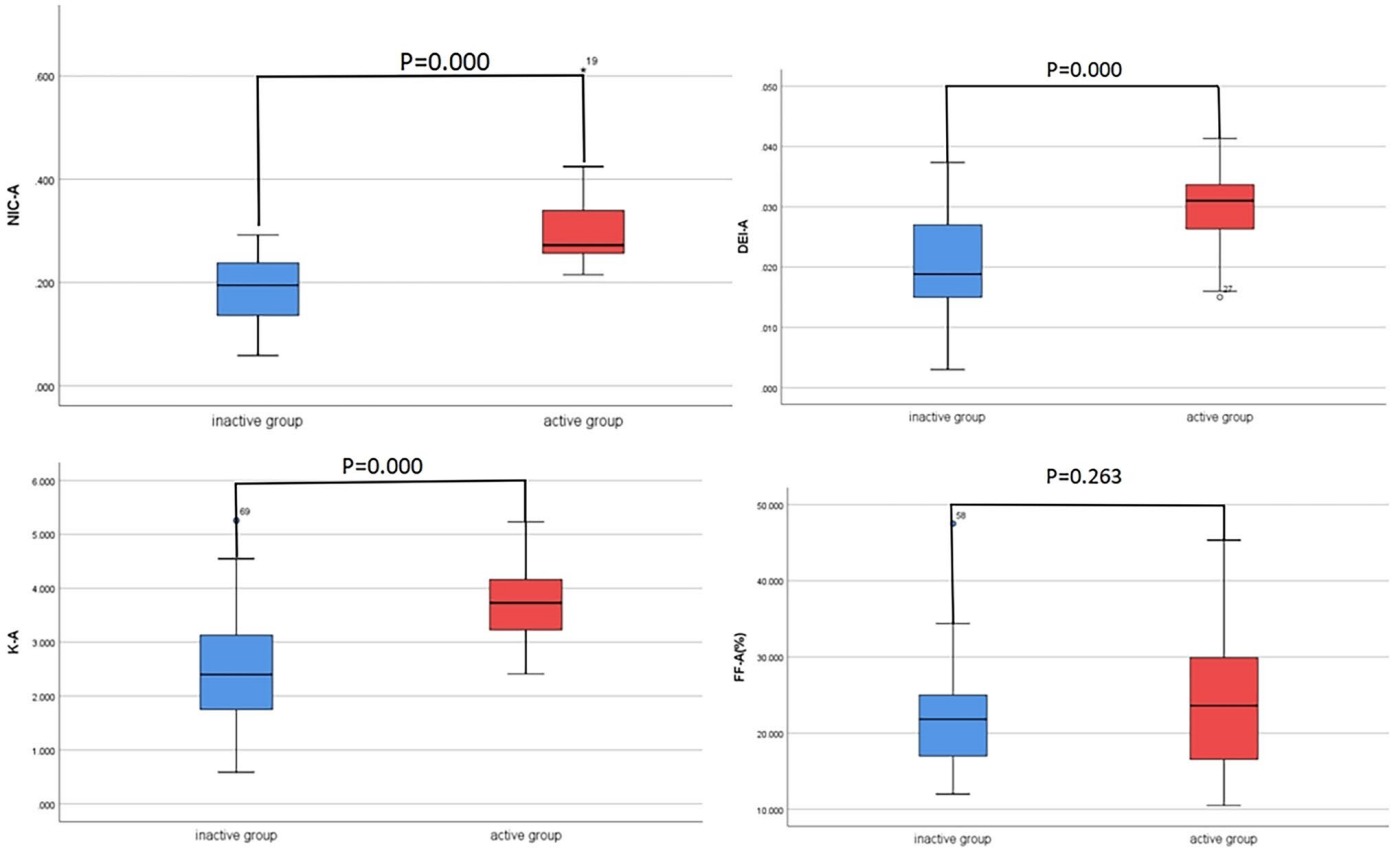
The NIC, energy-spectrum curve slope K and DEI of the active and inactive bowel segments in arterial phases were significantly different while the FF were not

**Fig. 2:**
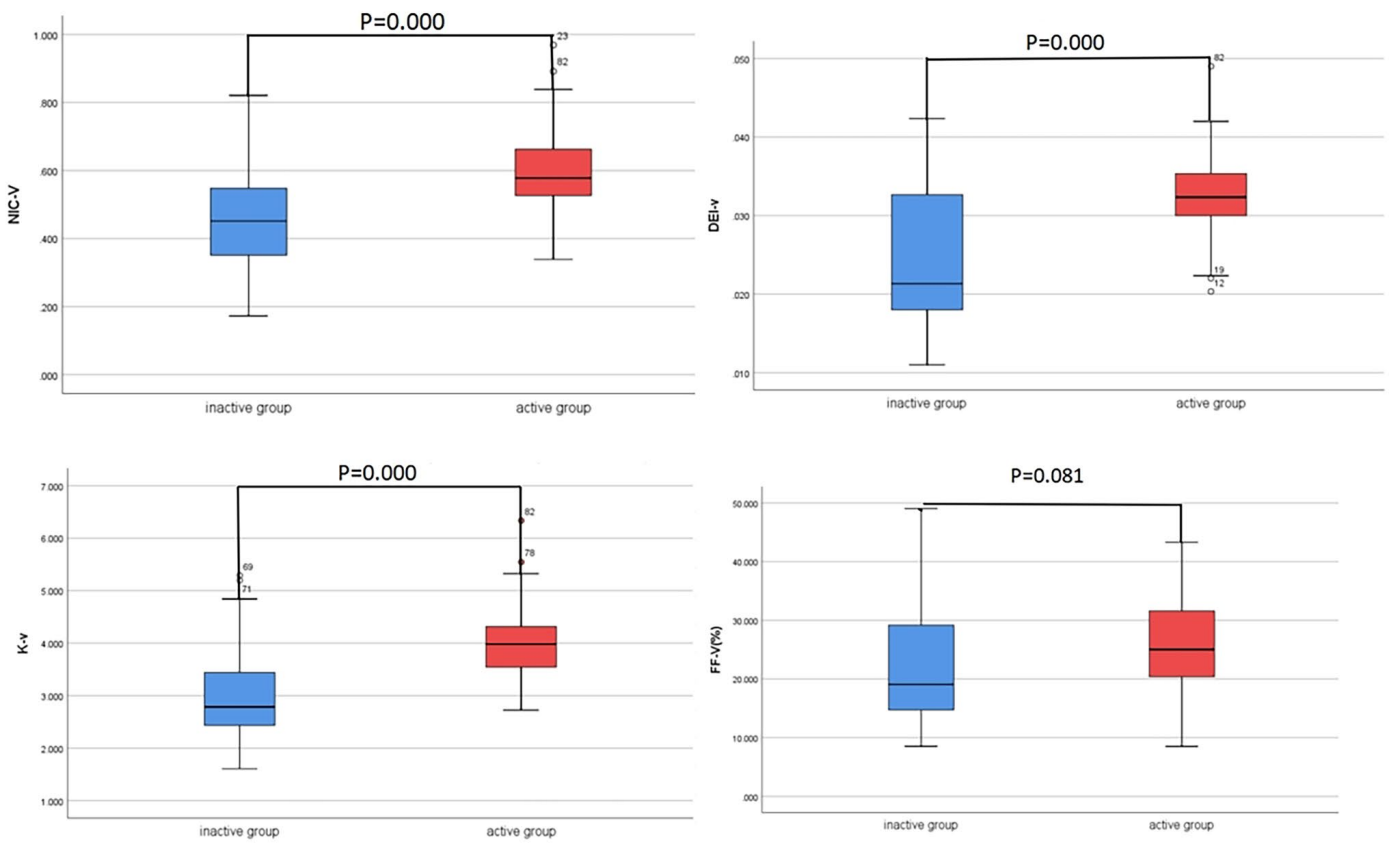
The NIC, energy-spectrum curve slope K and DEI of the active and inactive bowel segments in venous phases were significantly different while the FF were not

**Fig. 3:**
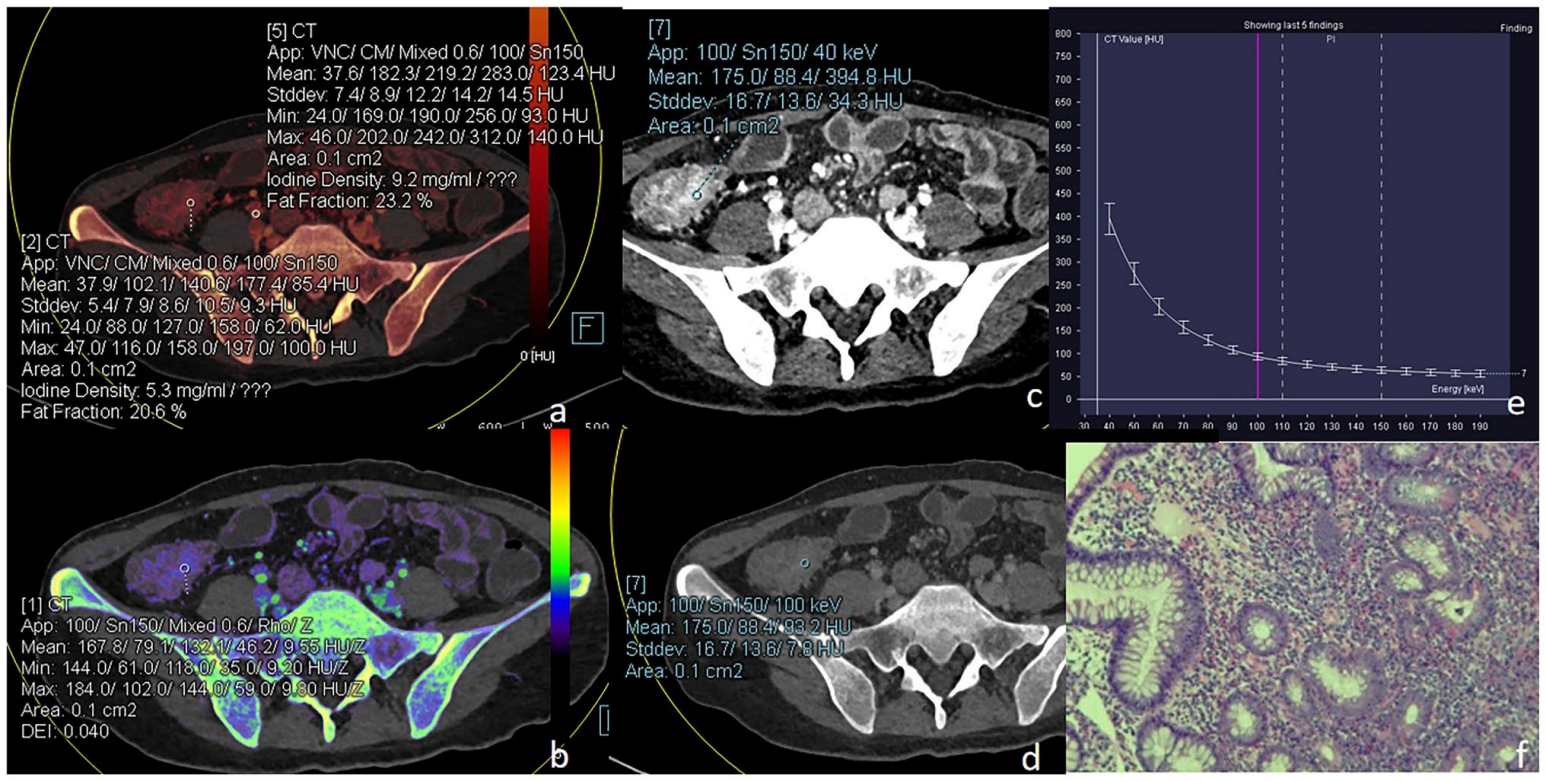
A patient with active CD, female, 27 years old, was admitted with recurrent abdominal pain for more than 10 years. **a** is an iodine map in venous phase showing a target segment iodine concentration of 5.3 mg/ml, FF of 20.6%, corresponding to an arterial iodine concentration at the same level of 9.2 mg/ml, and NIC of 0.576. **b** Shows the effective atomic number map with a DEI of 0.040. **c–e** Show the virtual single-energy maps and energy-spectrum curves at 40 and 100 keV, with CT values of 394.8 HU and 93.2 HU for the target intestinal segments, respectively, and a calculated K value of 5.027; **f** is a pathological image showing moderate chronic inflammation of the gyrus with ulcer formation and occasional cryptitis (HE × 100)

**Fig. 4:**
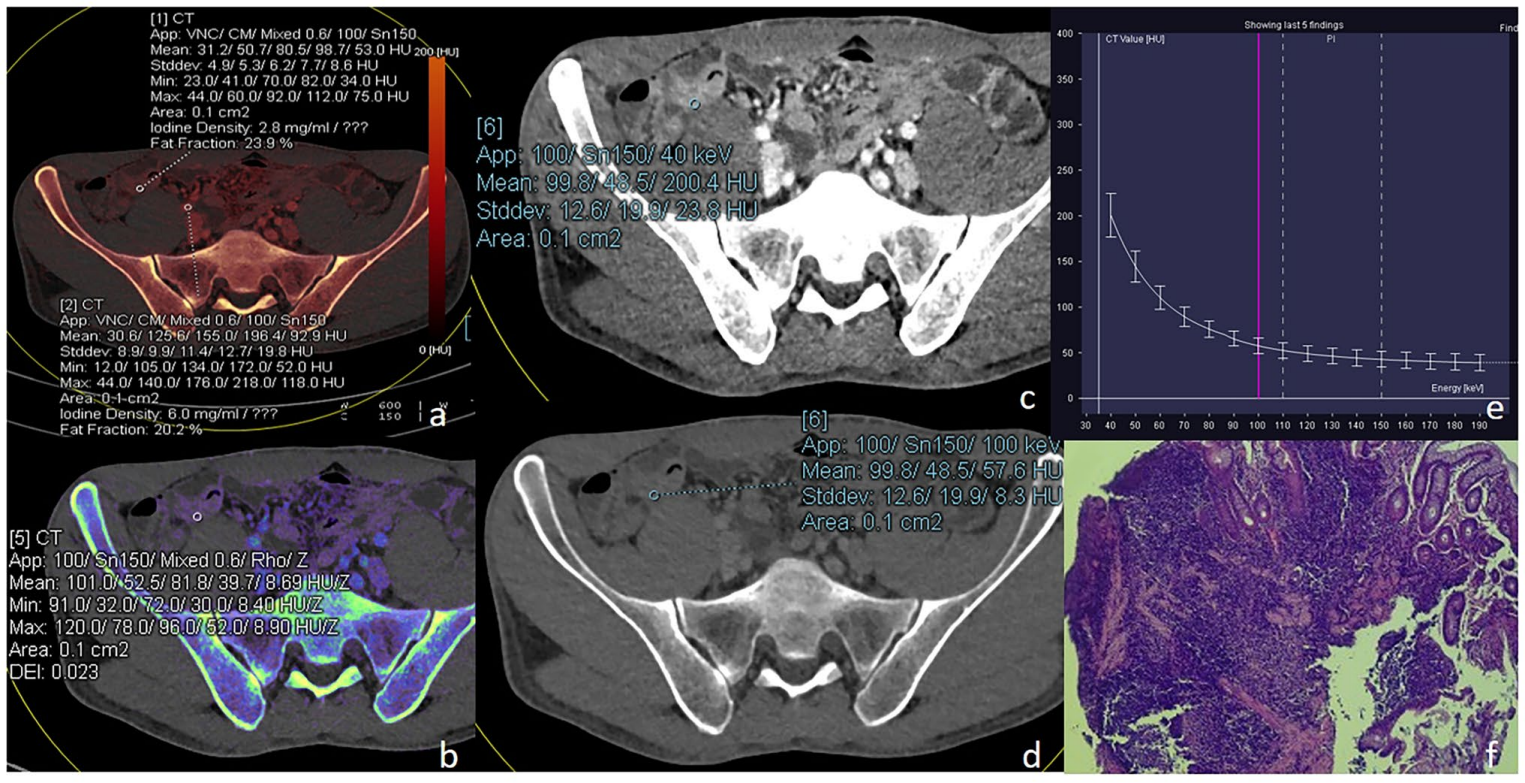
A patient with inactive CD, male, 32 years old, was admitted with diarrhea for 1 year. **a** is an iodine map in venous phase showing a target segment iodine concentration of 2.8 mg/ml, FF of 23.9%, corresponding to an arterial iodine concentration at the same level of 6.0 mg/ml, and NIC of 0.467. **b** shows the effective atomic number map with a DEI of 0.023. **c–e** show the virtual single-energy maps and energy-spectrum curves at 40 and 100 keV, with CT values of 200.4 HU and 57.6 HU for the target intestinal segment, respectively, and a calculated K value of 2.380; **f** is a pathological image showing chronic inflammation of the (terminal ileum) mucosa, with a nested arrangement of sheets of hyperplastic lymphoid tissue seen in the submucosa, focal lymphoid follicle formation, and extrusion of the focal tissue (HE × 100)

The measurements of the NIC, energy-spectrum curve slope K, DEI and FF obtained by the 2 physicians were in good agreement, with ICC values of 0.88, 0.84, 0.82 and 0.86, respectively. The NIC, energy-spectrum curve slope K and DEI of the active and inactive bowel segments in arterial and venous phases were significantly different (*P* < 0.05) while the FF were not (*P* > 0.05; Figs. 1, 2 and Table 2). NIC, energy-spectrum curve slope K, DEI, FF were higher in active bowel segments than in inactive ones, although there was no statistically significant difference in FF. The pathology, iodogram, energy-spectrum curve and DEI of patient in the active and inactive bowel segments of the venous phases are shown in Figs. 3 and 4, respectively.

**Table 2:** Comparison of NIC, energy-spectrum curve slope K, DEI, FF in arterial and venous phases

	Group	NIC	K	DEI	FF (%)
Arterial phase	Active group	0.272(0.256, 0.340)	3.735 ± 0.655	0.030 ± 0.006	23.707 ± 8.133
	Inactive group	0.186 ± 0.064	2.552 ± 1.109	0.021 ± 0.008	21.817(16.808, 25.142)
	T/Z	− 4.509	− 5.348	− 5.591	− 1.120
	P	0.000	0.000	0.000	0.263
Venous phase	Active group	0.592 ± 0.128	3.999 ± 0.713	0.032 ± 0.006	25.749 ± 8.245
	Inactive group	0.455 ± 0.150	2.786(2.400, 3.571)	0.021(0.018, 0.033)	22.230 ± 9.650
	T/Z	− 4.412	− 4.397	− 4.254	− 1.769
	P	0.000	0.000	0.000	0.081

*NIC* normalized iodine concentration; *DEI* dual energy index; *FF* fat fraction

**Fig. 5:**
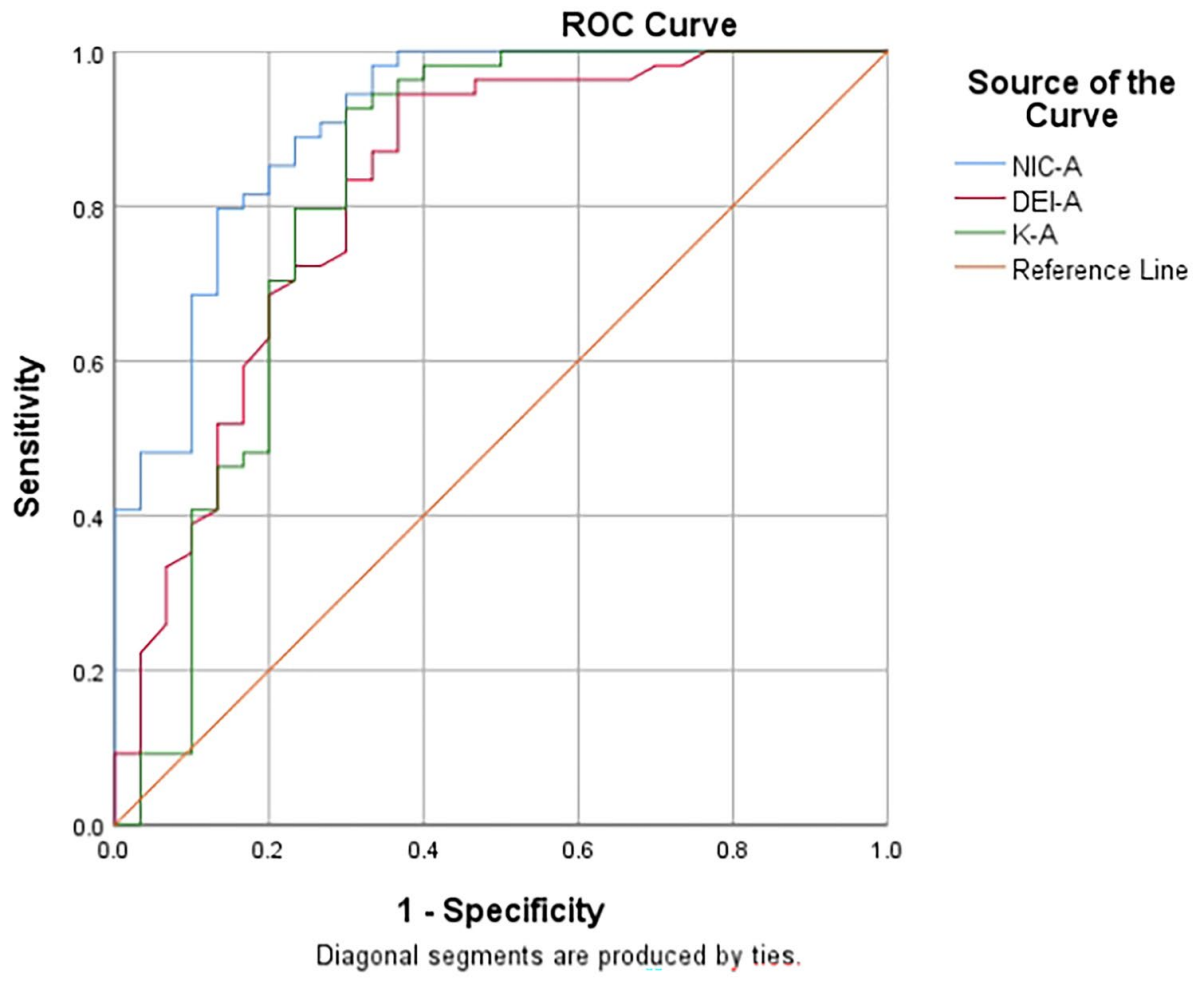
ROC curves for each parameter in arterial phase to identify active and inactive bowel segments in Crohn’s disease. The AUC of NIC, DEI and K in arterial phase were 0.908, 0.819 and 0.820, respectively

**Fig. 6:**
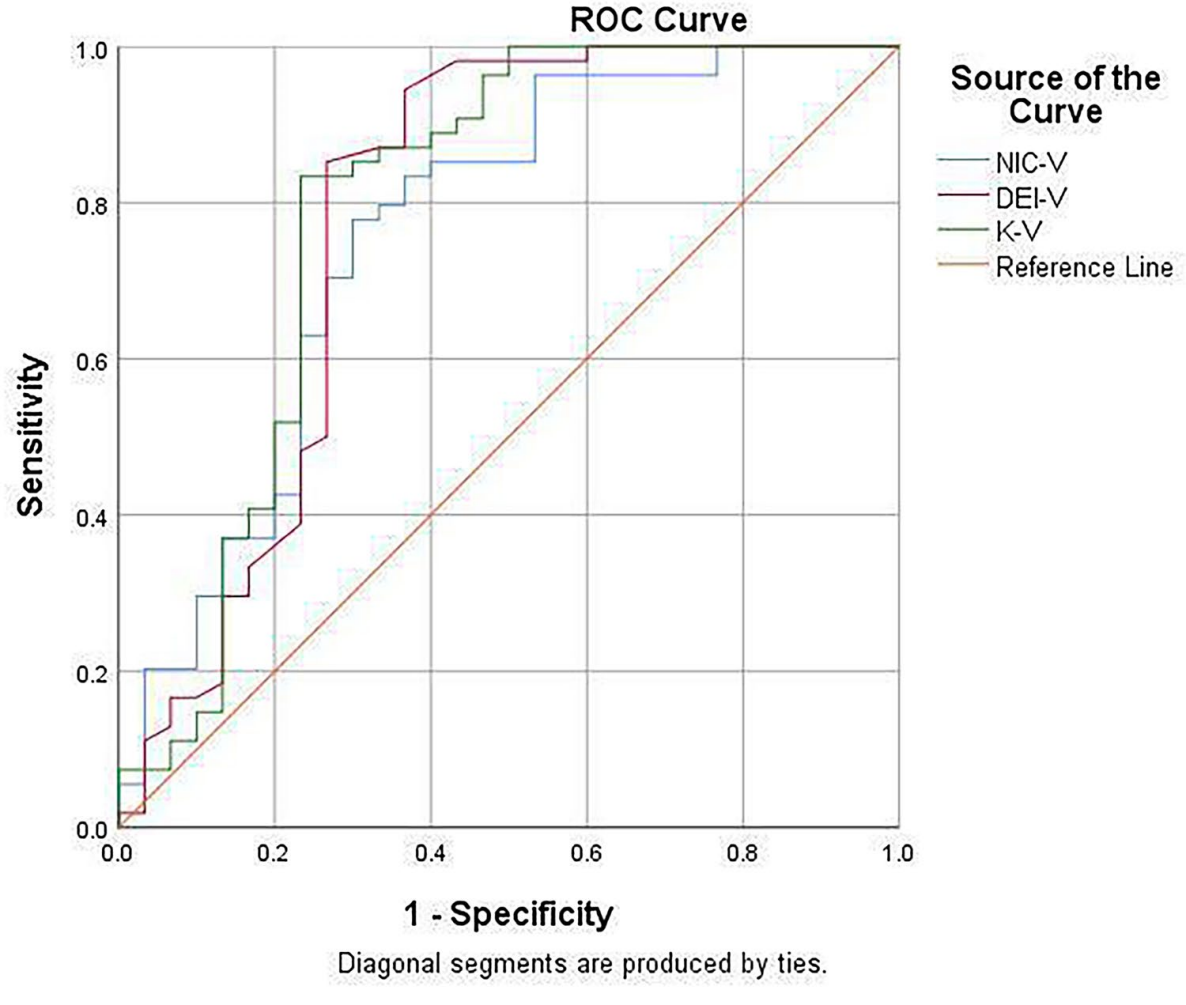
ROC curves for each parameter in venous phase to identify active and inactive bowel segments in Crohn’s disease.The AUC of NIC, DEI and K in venous phase were 0.760, 0.781 and 0.791, respectively

The ROC curves of arterial and venous phases were plotted respectively. The AUC of NIC, energy-spectrum curve slope K and DEI in arterial phase was higher than that in venous phase, and there was statistical difference between the AUC of NIC in arterial phase and that in venous phase. Among the parameters, the AUC of the NIC in arterial phase was the largest (Tables 3, 4 and Figs. 5, 6).

**Table 3:** Results of ROC curves for each parameter in the arterial and venous phases

	Parameter	AUC	AUC (95%CI)	Maximum Youden Index	Threshold	Sensitivity (%)	Specificity (%)
Arterial phase	NIC	0.908	0.841–0.975	0.633	0.245	0.833	0.8
	K	0.820	0.708–0.933	0.626	2.896	0.926	0.7
	DEI	0.819	0.717–0.921	0.577	0.021	0.944	0.633
Venous phase	NIC	0.760	0.646–0.875	0.478	0.522	0.778	0.700
	K	0.791	0.672–0.910	0.600	3.457	0.833	0.767
	DEI	0.781	0.621–0.881	0.585	0.027	0.852	0.733

*NIC* normalized iodine concentration; *DEI* dual energy index; *AUC* area under the curve; *CI* confidence interval

**Table 4:** Comparison of diagnostic efficiency of ROC curves of each parameter in arterial and venous phases

Parameter	95%CI	Z value	*P*
NIC	0.0265–0.269	2.389	0.0169
K	− 0.0345–0.0937	0.906	0.3648
DEI	− 0.0348–0.110	1.018	0.3087

*NIC* normalized iodine concentration; *DEI* dual energy index; *CI* confidence interval

## Discussion

There is no gold standard for the diagnosis of CD, which requires a combination of clinical manifestations, imaging, endoscopy and pathology. There are a variety of reference indicators for identifying whether it is in the active period, each of which has its own advantages and disadvantages. Dual-energy CT has 2 independent bulb-detector systems that can simultaneously obtain the attenuation coefficients of substances at different radiation energy levels, which not only enables the acquisition of conventional CT enterography findings, including increased bowel wall thickness, bowel wall hyperenhancement, comb sign and mesenteric fat infiltration, but also provides several parameters for diagnosis using the corresponding postprocessing workstations [12], including iodine content, virtual single-energy CT values, energy-spectrum curve slope K values, FF and DEI. DECTE scanning results in a shorter scanning time and reduced radiation dose relative to conventional CT scanning [13, 14]. In addition, direct measurement of iodine concentration using DECTE may be less dependent on scan parameters (manufacturer, energy, contrast material time) than conventional Hounsfield unit–based measurements [15]. This is important because patients often need to be imaged for years in different hospitals using different scanners.

Previous studies [5, 16] have demonstrated that dual-energy iodine content is a good surrogate marker for perfusion in various body imaging application. NIC was used in this study to minimize the effects of a variety of confounders, such as individual differences and contrast flow rates. Previous research [17] has suggested a strong correlation between iodine content and the CDAI or endoscopy findings. Xiao et al. [18] assessed iodine contents in the arterial and venous phases using DECTE to differentiate between active and inactive Crohn’s disease. Using the CDAI as a reference standard, at arterial phase iodine concentrations greater than 2.55 mg/mL, the reported specificity and sensitivity were 100% and 61%, respectively. In this study, using pathology as the reference standard, the NIC of CD patients significantly differed between the active and inactive phases. This was because of the inflammatory cell infiltration in the mucosa and the increased blood supply; a more severe inflammatory reaction is associated with a more obvious the blood supply increase and thus a higher iodine content [18]. This study suggests that the AUC of NIC in the arterial phase is the largest at 0.908, and when the NIC is greater than 0.245, the sensitivity of NIC for diagnosing CD intestinal segments in the active phase is 0.833, and the specificity is 0.800.

In addition, the energy-spectrum curve slope K and DEI were also significantly different between active and inactive bowel segments, and the energy-spectrum curve slope K and DEI of active bowel segments were higher than those of inactive bowel segments. Infact, both the NIC and the energy-spectrum curve slope K can be considered direct or indirect expressions of the iodine content within the ROI and thus should not be significantly different from each other [19]. In accordance with the use to distinguish active from inactive CD intestinal segments, Xiao et al. [18] found that energy-spectrum curve slope K could effectively distinguish normal from active intestinal segments in CD patients. DEI represents the difference between the X-ray attenuation coefficients of a substance at two different energies. Different substances have different, fixed DEIs; for example, water has a DEI of 0, and fat has a DEI of − 0.0242, but the values are affected by the contrast agent, and when the contrast agent is injected, the DEI increases and is directly proportional to the concentration of the contrast agent.

This study also assessed the FF, but the results showed that they were not significantly different between the active and inactive phases. Relatively few studies have been conducted on dual-energy FF, and Villanueva Campos [20] found that FF was significantly higher in normal small intestines than in inflammatory bowel segments. Isabelle De Kock et al. [21] in contrast, showed that the FF was unable to differentiate between normal small bowel and active CD. However, their studies did not have a reliable reference standard such as endoscopy, histologic samples. Indeed, submucosal fat accumulation in the bowel wall, known as the "fat halo sign" on CT imaging, has been described in long-term CD patients and is associated with chronic intestinal inflammation, which is not typical of active disease [22].

In this study, we found that the AUC of NIC, energy-spectrum curve K and DEI was higher in arterial phase than in venous phase, and the difference between the AUC of NIC was statistically significant. The reason for this may be due to the fact that the fibrosis of the inactive bowel wall in CD is more severe and often shows progressive enhancement, whereas the small arteries that are significantly dilated and tortuous in the arterial phase of the active bowel wall show reduced enhancement in the venous phase, resulting in a higher difference in iodine content between active and inactive bowel segments in the arterial phase than in the venous phase, which leads to a reduction in the difference in X-ray attenuation between the two in venous phase [23].

It is well known that CD is a chronic, nonspecific inflammatory disease with recurrent episodes that result in patients having both active and inactive bowel segments. However, commonly used clinical activity indices or biochemical indicators of Crohn’s disease, such as the Harvey-Bradshaw index and CDAI or FC and CPR, default to the same status for all diseased bowel segments, which does not correspond to reality. This is why these indicators do not always correlate with endoscopic or radiological findings [4, 16, 24]. The analyzes conducted in this study were based on the use of the pathological criteria of a particular bowel segment as a reference and thus are more specific to the diseased bowel segment itself, which is more consistent with real-world conditions. In addition, CDAI is more dependent on the subjective impression of the physician, whereas endoscopic disease activity, such as SESCD and CDEIS, can only be assessed for mucosal status, while submucosal and muscular diseases cannot be shown.

Of course, there are some disadvantages in this study: (1) The sample size of this study is small, and more patients need to be included in future studies; (2) We performed manual ROI analyzes of the diseased bowel segments, which inevitably resulted in some errors, and we need to develop software for automated or semiautomated segmentation of the bowel in the future; (3) Consistency within the observation group was not assessed; and (4) There may be imprecise anatomical correlation between the assessed endoscopic specimens and imaging.

## Conclusion

In this study, using pathology as a reference standard, parameters such as NIC, energy-spectrum curve slope K and DEI were obtained using dual-energy CT enterography, effectively allowing the differentiation between active and inactive bowel segments in patients with CD and providing good assistance for determining further treatments for patients. In addition, it showed better diagnostic efficacy in arterial phase than in venous phase.
